# Supplementary material for: Knowledge Domain and Emerging Trends in Vinegar Research: A Bibliometric Review of the Literature from WoSCC
Source: Foods. 2020 Feb 10;9(2):166. doi: 10.3390/foods9020166 (PMC7074530; doi:10.3390/foods9020166)
Supplement: Supplementary file 1 [file foods-09-00166-s001.zip › Supplementary files/Supplementary Material/Supplementary data.docx]

Knowledge Domain and Emerging Trends in Vinegar Research: A Bibliometric Review of the Literature from WoSCC

Xiang-Long Zhang, Yu Zheng, Meng-Lei Xia, Ya-Nan Wu, Xiao-Jing Liu, San-Kuan Xie, Yan-Fang Wu and Min Wang*

State Key Laboratory of Food Nutrition and Safety, Key Laboratory of Industrial Fermentation Microbiology, Ministry of Education, College of Biotechnology, Tianjin University of Science & Technology, Tianjin 300457, China; xl_zhang@mail.tust.edu.cn (X.-L.Z.); yuzheng@tust.edu.cn (Y.Z.); mlxia@tust.edu.cn (M.-L.X.); 18820944@mail.tust.edu.cn (Y.-N.W.); lxj9704@163.com (X.-J.L.); xskuan@163.com (S.-K.X.); amwyvonne@163.com (Y.-F.W.);

***** Correspondence: [minw@tust.edu.cn](mailto:minw@tust.edu.cn); Tel.: +86-22-60600045

*(Supplementary data)*


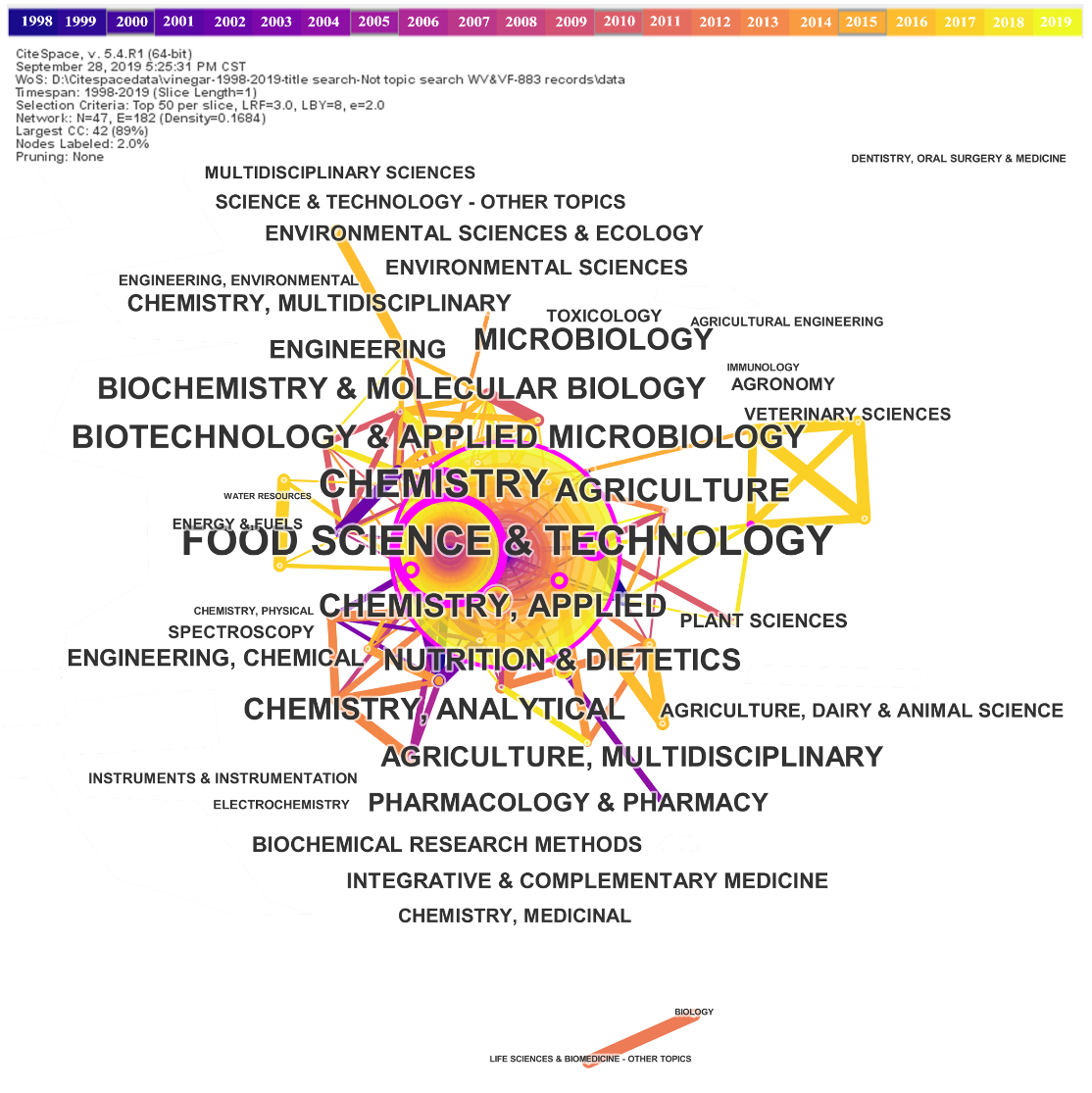


**Figure S1.** The subject category network for vinegar research from 1998 to 2019 based on JCR categories at WoS.


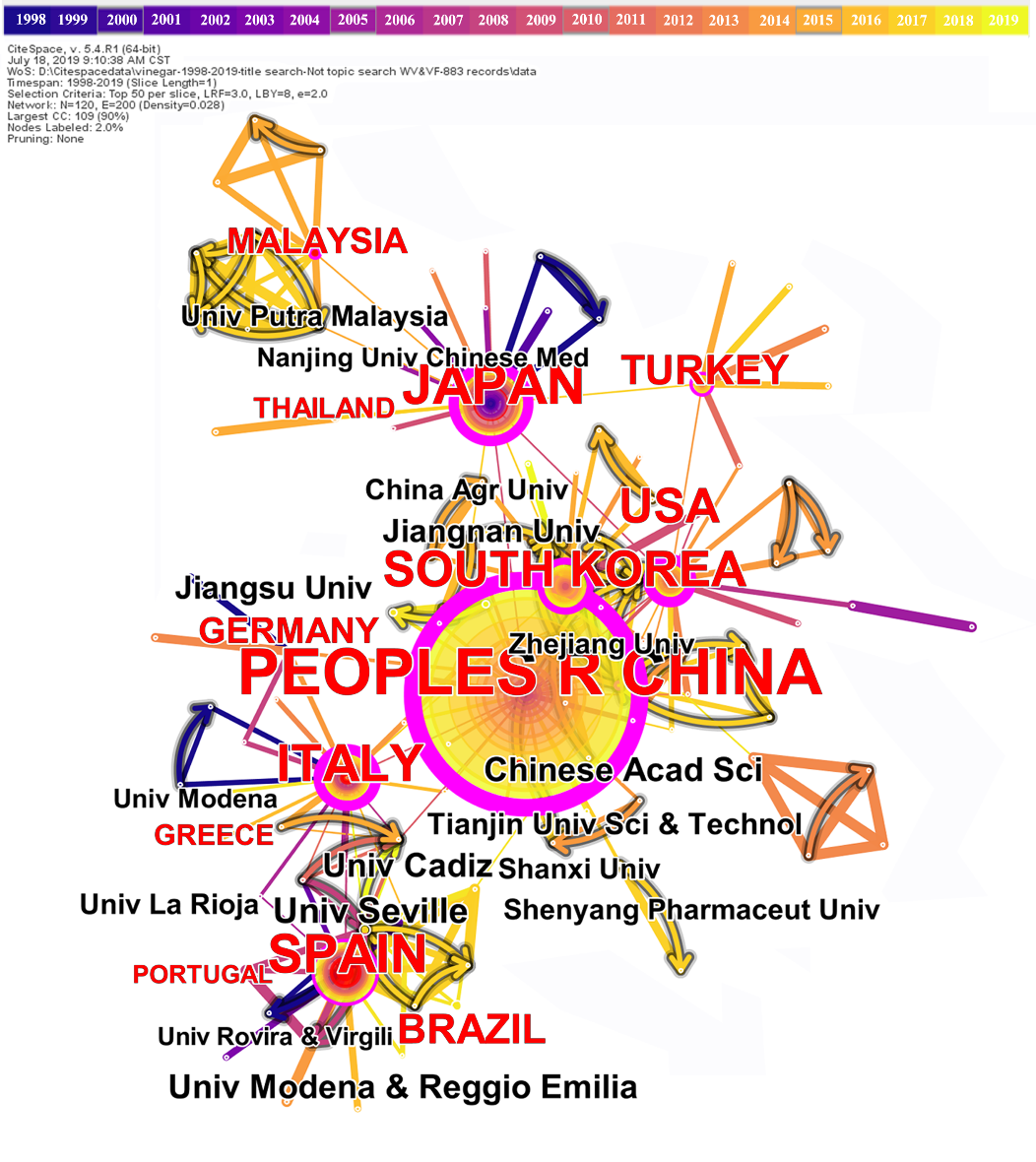


**Figure S2.** The network map of countries and institutes for vinegar research.
